# Chromosomal Aberrations in Cattle

**DOI:** 10.3390/genes12091330

**Published:** 2021-08-27

**Authors:** Beáta Holečková, Viera Schwarzbacherová, Martina Galdíková, Simona Koleničová, Jana Halušková, Jana Staničová, Valéria Verebová, Annamária Jutková

**Affiliations:** 1 Department of Biology and Physiology, University of Veterinary Medicine and Pharmacy, Komenského 73, 041 81 Košice, Slovakia; viera.schwarzbacherova@uvlf.sk (V.S.); martina.galdikova@uvlf.sk (M.G.); simona.kolenicova@uvlf.sk (S.K.); jana.haluskova@uvlf.sk (J.H.); annamaria.jutkova@uvlf.sk (A.J.); 2 First Faculty of Medicine, Charles University in Prague, Salmovská 1, 121 08 Prague, Czech Republic; jana.stanicova@uvlf.sk; 3 Department of Chemistry, Biochemistry and Biophysics, University of Veterinary Medicine and Pharmacy, Komenského 73, 041 81 Košice, Slovakia; valeria.verebova@uvlf.sk

**Keywords:** chromosomes, aberrations, Robertsonian translocations, reciprocal translocations, genotoxic agents, cattle

## Abstract

Chromosomal aberrations and their mechanisms have been studied for many years in livestock. In cattle, chromosomal abnormalities are often associated with serious reproduction-related problems, such as infertility of carriers and early mortality of embryos. In the present work, we review the mechanisms and consequences of the most important bovine chromosomal aberrations: Robertsonian translocations and reciprocal translocations. We also discuss the application of bovine cell cultures in genotoxicity studies.

## 1. Introduction

Cattle (*Ruminantia*, *Bovidae*) have been closely associated with humans from prehistoric times. They were first domesticated from wild cattle (*Bos primigenius*) in the Middle East about 8000–10,000 years ago [1] or more precisely in the 9th millennium BC in Southwest Asia [2].

At present, domesticated cattle (*Bos taurus*) offer a significant source of nutrition and livelihood to the human population almost all over the world. Besides that, breeds of cattle represent an important world heritage and provide scientific resource for study of economically important traits, such as metabolism, lactation, reproduction, disease resistance as well as for understanding the genetics of complex traits [3]. Unique anatomical and physiological characteristics led to the sequencing of the cattle genome [4], reporting at least 22,000 genes and 14,345 orthologs shared among seven mammalian species. It also has transposable element classes similar to other mammals as well as large numbers of ruminant-specific repeats that comprise 27 percent of its genome [3]. The Bovine Genome Database (BGD; http://BovineGenome.org, accessed on 8 March 2021), reported by Childers et al. [5], strives to improve annotation of the bovine genome and to integrate the genome sequence with other genomics data. The group of Stothard et al. [6] elaborated the Canadian Cattle Genome Project. The aim of the project was the developing of genomics-based tools to enhance the efficiency and sustainability of beef and dairy production.

The latest Bovine Genome Database (BGD; http://bovinegenome.org, accessed on 8 March 2021) is a web-accessible resource that supports bovine genomics research by providing genome annotation and data-mining tools [7].

The genome sequencing project should open new opportunities for research, including the animal cytogenetic field. Despite the rapid development of new molecular techniques (array platforms, next-generation sequencing), chromosome analysis remains a key procedure for screening of chromosomal aberrations in animal cytogenetic laboratories. This is also possible due to fluorescence in-situ hybridization (FISH) that still represents an important diagnostic and research tool in bovine chromosome and genome analysis.

## 2. Animal Cytogenetics

**Figure 1:**
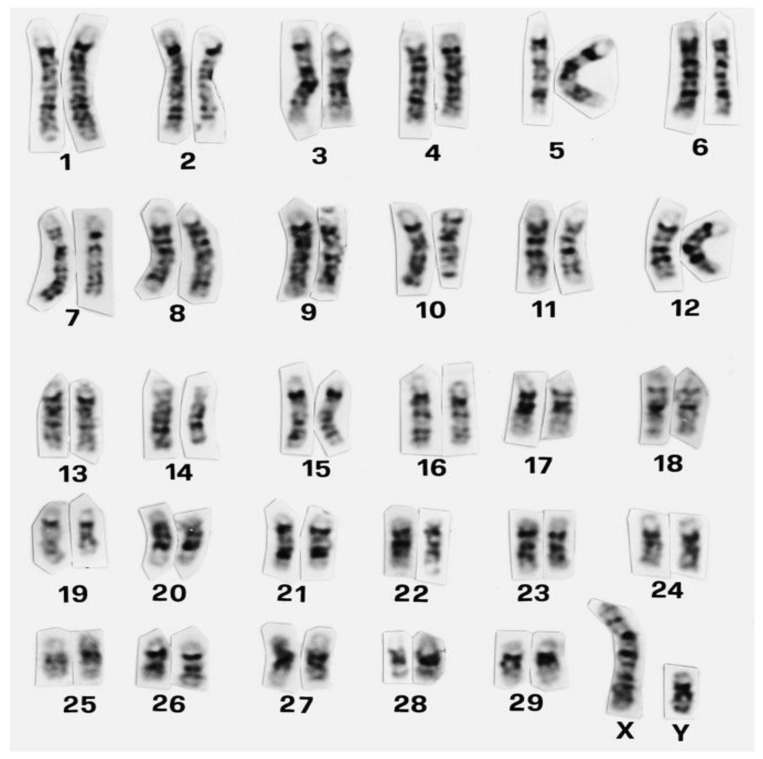
Standard GTG-banded karyotype of cattle (Cribiu et al., 2001). Reprinted with permission from ref. [11]. Copyright 2001 S. Karger AG.

The first European meeting on cytogenetics of domestic animals was held in 1970 [8]. From the very beginning of animal cytogenetic research, cattle and pigs attracted the attention of cytogenetics. In cattle alone, over 13,000 animals belonging to 80 different breeds were karyotyped by the middle of 70s, with the largest contributions by Sweden, Germany, and France [9]. The establishment of international standard karyotypes for domestic mammals including cattle was facilitated by the discovery of chromosome-banding techniques (e.g., method named G-banding) (Figure 1) that were elucidated in detail by Iannuzzi and Di Berardino [10]. Banding methods allow correct identification of individual chromosomes and their precise arrangement into specific homologous pairs in the karyotype (Figure 1). This is valuable also for a more specific description of chromosomal aberrations if they occur. For instance, karyotype of cattle is composed of 58 acrocentric autosomes and two subtelomeric sex chromosomes (2n = 60, XX or 2n = 60, XY) [11]. The interesting feature of cattle karyotype is that it represents the ancestral type. It means that in *Bovidae*, other species’ karyotypes evolved via Robertsonian fusions of ancestral acrocentric chromosomes [12,13]. A recent attempt of the application of chromosome-banding techniques (C, G, and NOR) was their use in determining the karyotype characteristics of the Nelore Brasilian breed of cattle *(Bos taurus indicus* Linnaeus 1758) [14]. However, the results of the banding methods used here suggest that these methods need to be applied more precisely in order to obtain a good-quality image of the chromosome regions.

At present, standard karyotypes and chromosome nomenclatures are available for several domestic species; in most of them, chromosomal aberrations and their breakpoints might be retraced to the DNA sequence level because individual chromosomes are aligned with the species reference genome sequence [15].

## 3. Chromosomal Aberrations

### 3.1. Classification of Chromosomal Abnormalities

In general, chromosomal abnormalities can be classified into numerical aberrations (euploidy, such as monoploidy, polyploidy, and aneuploidy−monosomy, trisomy) as well as structural rearrangements [16]. Numerical aberrations of the *autosomes* (polyploidies and aneuploidies) are typically lethal at the embryonic stage and rarely found in live-born individuals [15]. This is probably due to eliminations in early embryonic development or by breeders when severe anatomical defects occur [17]. In addition, low levels of sperm aneuploidies in bulls can play a role.

Polyploidy is the result of abnormal fertilization (polyandry or polygyny): suppression of the first cleavage division in embryogenesis or fusion of embryonic cells [18]. Aneuploidy arises from the nondisjunction of homologous chromosomes during meiosis by unbalanced chromosome segregation in meiosis (anaphase I or anaphase II) in animals. The result is the presence of a small proportion of unbalanced gametes among normal ones; their fertilization leads to the development of embryos with abnormal chromosomal complement, which usually die during early prenatal development. In general, the embryonic lethal effect could be explained by the loss or excess of chromosomal genetic material involved in the specific aberration. For example, in a euploid, the ratio of genes on any one chromosome to genes on other chromosomes is 1:1 (that is, 100 percent), regardless of whether a monoploid, diploid, triploid, or tetraploid are considered. In contrast, in an aneuploid, the ratio of genes on the aneuploid chromosome to genes on the other chromosomes differs from wild type by 50 percent (50 percent for monosomics; 150 percent for trisomics). Thus, the aneuploid genes are out of balance (disruption of gene balance occurs) [19]. Consequently, the relative dosage of certain genes changes and physiological imbalances in cellular pathways can be present. Taking into consideration the previous facts, the aneuploid phenotype (the aneuploid phenotypic syndrome) is very probably a complex of the imbalance effects of a few major genes together with a cumulative imbalance of many minor genes. According to Raudsepp and Chowdhary [15], the rare viable cases with numerical aberrations usually show multiple and severe congenital malformations and have primary infertility, preventing the aberrations from being transmitted to offspring. An example can be the trisomy of chromosome 28 revealed in a one-year-old Hereford female calf with slow growth, brachygnathia superior, hyper-salivation, strabismus convergence, macroclitoris, a duplication of the uterine cervix, and other defects [20]. Similarly, trisomy of chromosome 18 associated with extreme brachygnathia (lethal brachygnathia trisomy syndrome, LBTS) has been reported in calves that died soon after birth [21]. Trisomies of several other smaller chromosomes, such as 12, 16, 17, 20, 22, 23, 24 [21], and chromosome 29 [22], have been detected in cattle with severe anatomical abnormalities mostly resulting in early death (recently overviewed in Iannuzzi et al. [23]). In 2015, partial trisomy 25q and partial monosomy 11q (60, XX) were detected in a newborn calf of Agerolese cattle who died after two weeks. The calf, whose mother carried a chromosomal aberration, underwent cytogenetic investigation because of hyperflexion of the forelimbs, red eyes, and the inability to stand [24]. The authors performed a comparison with human chromosomes to search for similarities and possible genes involved. They did not identify similarities with human diseases but concluded that further molecular investigation at the gene level would be of interest to find the cause for the aberration reported in the calf.

In conclusion, trisomies usually involve smaller chromosomes rather than larger ones. The cause of autosomal trisomies is non-disjunction during gametogenesis in one of the parents or during early cell division in the fertilized egg.

When compared with autosomes, aneuploidies of sex chromosomes are more frequent. They have a milder effect on viability but a negative impact on fertility [25,26,27]. Sex chromosome abnormalities, such as X trisomy and X monosomy, XXY syndrome, sex reversal syndromes (XY; XX), and chimerism XX/XY, are described in the latest work of Iannuzzi et al. [23]. The last-mentioned syndrome is the most common sex chromosome abnormality observed in bovine twins of different sexes, connected with reduced fertility of females and to a lesser extent in males. The reason is a placental anastomosis between embryos and the time schedule of embryonic male sex differentiation. This occurs one week before female sex. The presence of male cells and hormones (anti-Müllerian hormone, AMH) influences the development of female sex characteristics.

Recently, a unique mosaic karyotype with a small marker chromosome (autosome) (60, XX/60, XX, +mar) was identified in a Holstein-Fresian calf with multiple congenital malformations [28]. It can be supposed that the presence of the small marker autosome may be associated with observed congenital malformations in the studied calf.

Structural aberrations are typically caused by mistakes in meiotic recombination and DNA-break repair [15]. This aberrations can be divided into unbalanced, which changes the DNA content (deletions, duplications, insertions, isochromosome), and balanced, which does not change the DNA content (translocations and inversions). Large unbalanced rearrangements usually cause early embryonic death, while balanced structural rearrangements impair fertility due to mortality of embryos with an unbalanced chromosome complement [16]. In humans, both types of rearrangement (unbalanced and balanced) have been shown to have impacts on gene expression through a variety of different mechanisms [29]. It was observed that a few to several percent of translocations disrupt haploinsufficient genes or their regulatory regions and result in clinical phenotypes [30].

### 3.2. Robertsonian Translocations in Cattle

As indicated, reproduction-related problems in cattle are at the centre of breeders’ interest. They are often associated with chromosomal abnormalities, which result in infertility of carriers and early mortality of embryos. In some cases, also the mortality of newborns, degeneration of reproductive organs, poor semen quality, and lower body mass increase in the offspring are observed.

**Figure 2:**
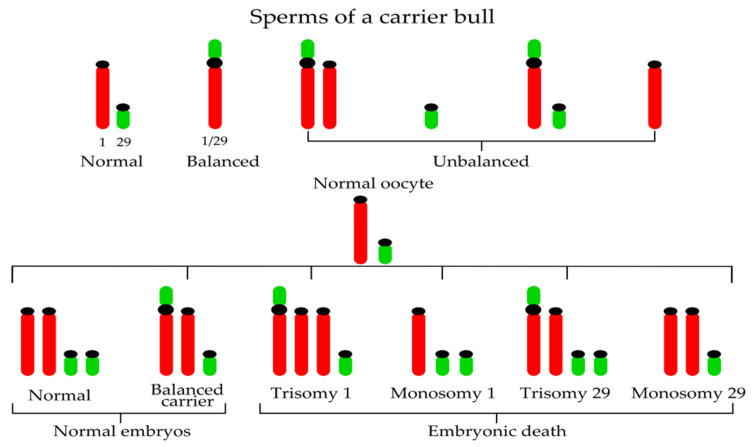
Examples of chromosomal combinations in embryos resulting from the mating of a heterozygous carrier bull 1;29 and a normal cow.

The most commonly detected chromosome change in cattle is the so-called Robertsonian translocation or centric fusion, where two acrocentric chromosomes break and fuse at the centromeric region. On the basis of specific chromosomes involved, approximately 44 different types of Robertsonian translocations (rob) have been described in cattle up until 2015 [18]. Among them, the Robertsonian translocation involving chromosomes 1 and 29—rob(1;29)—is the most widely spread across different breeds [31] (Figure 2) and has so far been identified in more than 50 breeds worldwide [32]. However, some authors observed differences in the prevalence of rob(1;29) among breeds (Table 1), with the frequency reaching up to 60% in British White and Corsican breeds [33,34].

For the first time, the 1;29 Robertsonian translocation was reported by Gustavsson and Rockborn in Swedish Red and White cattle in 1964 [35]; subsequent field studies showed a 13–14% incidence of the translocation in the population. Later, Gustavsson [36] identified an unequivocal association between heterozygosity for the 1;29 translocation and a 4–5% reduction in the fertility of the breed. He demonstrated that not only do chromosome abnormalities occur in domestic livestock, but they also have physiological effects on carrier animals with economic consequences [37] associated particularly with reproductive problems. Fertility reduction in carrier heterozygous bulls is caused by the production of unbalanced gametes (approximately 3.22% unbalanced sperms in a carrier bulls [38]) during meiosis (nullisomy or disomy for the chromosomes involved in the translocation) (Figure 2).

**Figure 3:**
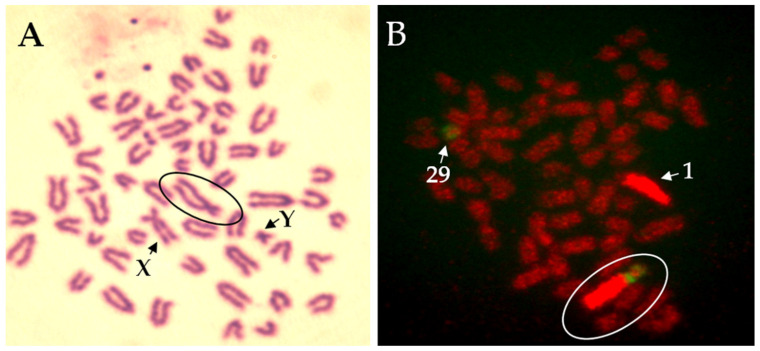
Robertsonian translocation 1;29 in bull (Slovak spotted breed). (**A**) Giemsa staining of chromosomes. (**B**) Fluorescence in-situ hybridisation (FISH) with whole chromosome painting probes for bovine chromosome 1 (BTA1, red) and 29 (BTA29, green). Circle indicates a fusion.

In other words, the formation of the trivalent and abnormal segregation of this trivalent at meiotic anaphase I is the reason for decreased fertility of centric fusion carriers. If a normal egg (from a noncarrier cow) is fertilized by the sperm of a carrier bull, two-thirds of embryos will possess unbalanced genetic material (trisomy BTA1, monosomy BTA1, trisomy BTA29, and monosomy BTA29) (Figure 2). Such autosomal imbalance causes early embryonic death and therefore the reduction of fertility. Considering one-third of normal embryos, 50% are noncarriers and 50% carriers of rob(1;29). Consequently, the daughters of carrier heterozygous bulls have reduced fertility when compared with daughters of normal bulls. For this reason, selective elimination of bulls carrying the translocation from use in artificial insemination is important (Figure 3). As commented by De Lorenzi et al. [39], when compared with other Robertsonian translocations, rob(1;29) would be of ancient origin because a de-novo origin has never been reported.

The results of Joerg et al. [40] suggested that the chromosomal rearrangements leading to rob(1;29) occur in or next to the centromeric alpha-repeat region, but the genomic structure of this anomaly has been only recently described by De Lorenzi et al. [39]. The authors demonstrated that during the fusion process, around 5.4 Mb of the pericentromeric region of BTA 29 moved to the q arm close to the centromere of rob(1;29) and that this fragment was inverted.

In addition to translocations 1 and 29, which are monocentric, there are many other types of dicentric Robertson translocations, as reviewed in [18,23]. Rob(1;21), rob(23;26), rob(24;26), and rob(26;29) were reported by Arslan et al. [41] in Holstein cattle—cows with Repeat Breeder Syndrome usually connected with the infertility problem (Table 1). A finding of a new dicentric Robertsonian translocation rob(3;16) in a bull from the Montbéliarde dairy cattle breed with a significant interchromosomal effect (ICE) for two different autosomes, BTA17 and BTA20, was recently published [38].

**Table 1:** The frequencies of Robertsonian translocation carriers in some breeds of cattle.

Chromosomes Involved in Rob Translocation (Centric Fusion)	Breed of Cattle	Frequency	Year	Reference
rob(1;29)	Swedish Red and White cattle	13−14%	1964	[35]
	Over 50 breeds of cattle		1964−2014	[31,32]
	British White	Up to 60%	1975	[33]
	Corsican	Up to 60%	1984	[34]
	local Portuguese cattle	above 50%	2008	[42]
	Maremmana	18.8%	2008	[26]
	Romagnola	13.0%		
	Podolian cattle	11.7%		
	Marchigiana	11.7%		
	Chianina	1.4%		
	Limousine	12.3%	2008	[26]
	blonde d´Aquitaine	7.9%		
	Charolaise	1.2%		
	Rubia Gallega	21.9%	2008	[26]
	Retinta	16.1%		
	Czech Simmental	27.08%	2009	[27]
	Andalusian breeds: Negra Andaluza	19.45%	2013	[43]
	Berrenda en Negro	28.9%−32.6%		
	Criollo	12.3%	2015	[44]
	Swiss American	7.5%		
	Braunvieh (Swiss Brown)	1.4%		
	Holstein	0.4%		
rob(1;21) rob(23;26) rob(24;26) rob(26;29)	Holstein	6.4% together	2016	[41]
rob(3;16)	Montbéliarde	Referred for the first time in one animal (bull)	2018	[38]
rob(13;23)	Ukrainian Red-and-Motley	1.9%	2019	[45]

The ICE could be probably caused by the general disorganization of the meiotic spindle during metaphases I and II. Therefore, misalignment of the chromosomes at the equatorial plate occurred.

For this reason, ICE should also be considered when assessing the putative effect of Robertsonian translocation on reproduction. Using the sperm-FISH methodology, the authors found the 5.87% overall rate of genetically unbalanced gametes, which originated mainly from adjacent segregation (5.41%) at the end of meiosis I. In 2019, a new Robertsonian translocation rob(13;23) was described in Ukrainian Red-and-Motley dairy cattle breed during screening of impaired fertility of cows [45]. The cow with this translocation gave one phenotypically healthy calf; however, the second calving ended with a miscarriage. These above-mentioned cases point to the importance of cytogenetic and molecular genetic testing of every elite male and female carrier that should be part of strategies for controlling genetic diseases [46].

### 3.3. Reciprocal Translocations in Cattle

When compared with Robertsonian translocations, reciprocal translocations (rcps) reported in cattle are relatively rare [16]. This could be caused by the inability of conventional Giemsa staining to visualize the rcps, by the absence of abnormal phenotype in carriers, or by the early death of embryos carrying rcp that contains genes with relevant functions in foetal development [47]. Approximately 20 reciprocal translocations have been described in cattle so far (overviewed in [18,23,24,48]). Recently, the study of Iannuzzi et al. [23] provided a comprehensive review of reciprocal translocations found to date in cattle, with the chromosomes involved and phenotype effects. As summarized by authors, rcps were found mostly in bulls with reduced fertility, such as subfertile ones, azoospermic or with rare spermatozoa, bulls with no libido, or testosterone-negative bulls. Less frequently, rcp were revealed in cows (dams) with reduced fertility and sporadically in calves.

As already mentioned, one important reason for the rare observation of rcps is probably that they escape standard cytogenetic analyses of Giemsa-stained chromosomes or banding procedures. Only 16% of reciprocal translocations can be detected using simple Giemsa techniques [48]. For this reason, their real frequency in breeds seems to be underestimated. The precise study using the mathematical and bioinformatics approach [48] showed that the expected frequency of reciprocal translocations in cattle is about four times higher than dicentric Robertsonian translocations. One of explanation is that the available banding techniques are not able to identify small chromosomal region exchanges as subtelomeric reciprocal translocations. Therefore, a combination of different approaches, such as banding techniques (RBG-banding, C-banding), FISH analysis (using bovine BAC probes and whole-chromosome probes), and array-CGH analysis, should be more suitable for better rcp characterization [49,50,51]. Some of the breakpoint regions involved in balanced reciprocal translocations can be gene rich, which was demonstrated by De Lorenzi et al. [47]. At least 200 genes were localized in the regions of rcp(9;11)(q27;q11), indicating that identification of the sequences disrupted by the breakpoints and verification of their consequences on rcp carrier phenotype may be a challenge for future investigation.

Recently, Jennings et al. [52] chose a new approach to accurate detection of Robertsonian and reciprocal translocations in cattle, using a multiple-hybridisation detection strategy. They developed a method that uses a panel of subtelomeric fluorescence in-situ hybridisation probes on a multihybridisation device as a means of highlighting the ends of each chromosome. This highlighting (visualising each end of every cattle chromosome) facilitates the identification of rearrangements between chromosomes.

Reciprocal translocations are characterized by material exchange between non-homologous chromosomes as a consequence of break-points on two or more different chromosomes [49]. If they are balanced, there is no loss of genomic material. On the contrary, for unbalanced rcps, the loss of a variable amount of genomic material is typical. As indicated by Switonski et al. [53], reciprocal translocations are responsible for serious economic consequences since carriers produce unbalanced gametes and consequently embryos with high probability of dying. If animals carrying a reciprocal translocation are considered, they have a normal phenotype, but their fertility is reduced. The reduction in fertility in animals heterozygous for rcp arises during meiosis I (anaphase I). To allow all homologous regions to synapse, four chromosomal structures have to come together and create quadrivalent. There are many possible outcomes of disjunction from a quadrivalent depending on how close the breakpoints are to the centromeres and on the type of the disjunction [21]. Consequently, at least some unbalanced gametes are produced containing one or more additional chromosomal segments or lacking some segments of chromosomes. When an unbalanced gamete combines with another gamete to form a zygote, that zygote is unbalanced and dies. This is why unbalanced gametes result in embryonic death.

A practical example of how balanced reciprocal translocation of mother gives rise to an unbalanced karyotype of a newborn calf incompatible with life was elucidated by Iannuzzi et al. [24]. The authors underlined the abnormal gametogenesis in a phenotypically normal mother carrying the rcp(11;25), giving rise to four types of unbalanced zygotes after fertilization with a normal bull sperm. These zygotes with unbalanced translocations end up as aborted embryos or with the early death of newborns.

The above facts suggest the importance of cytogenetic analysis in both potential mothers and bulls before inclusion in artificial insemination.

### 3.4. Approaches in Translocations Detection

Unfortunately, acrocentric morphology of bovine autosomes does not make easy the identification of specific chromosomes involved in the fusion. This is especially in case of centric fusion translocations if chromosomes with the similar length or small acrocentric chromosomes are included in the fusion [54]. The precision of identification of frequently occurring centric fusion translocations in cattle can be considerably increased using fluorescence in-situ hybridisation (FISH) either with whole-chromosome (or whole-arm) painting probes or with bovine specific Bacterial Artificial Chromosomes (BAC) probes.

Whole-chromosome or region-specific paint probes (“paints”) are collections of labelled DNA sequences derived from a specific type of chromosome or chromosomal segment [55]. Generally, the paints can be prepared by flow sorting multiple copies of specific chromosomes, which is followed by degenerate oligonucleotide-primed PCR (DOP-PCR) amplification. Cattle chromosomes, however, show poor separation in bivariate flow cytometry [56]. Therefore, laser microbeam microdissection and laser pressure catapulting procedures completed with DOP-PCR were preferred by Kubickova et al. [57] and lately by Frolich et al. [58] for the construction of chromosome-specific painting probes in cattle. In farm animals, more work must be done to make chromosome-specific molecular probes better commercially available. The most frequently available bovine commercial probes are whole-chromosome 1 and 29 painting probes for detection of the most common centric fusion in cattle and for identification sex chromosomes X and Y. Whole-bovine-chromosome painting probes for visualization of other chromosomes of cattle have gradually been prepared by some research groups [57,59,60]. De Lorenzi et al. [61,62] applied fluorescent in-situ hybridisation using bovine-specific BAC probes to confirm that the new centric translocations rob(14;17) and rob(21;23) represented a fusion of small acrocentric chromosomes. With banding techniques, specific BAC probes represent a relatively easy and reproducible method to confirm the chromosomes involved in cytogenetic anomalies. In general, BAC clone is usually a modified F-plasmid containing a DNA sequence (of approximately 30,000–300,000 bp) and a resistance gene. BACs are locus-specific probes: they can be applied not only to visualize translocations but also to identify deletions or duplications of the target region on a chromosome. They also serve for the location and characterization of the breakpoint regions [63]. BAC clones can be selected from BAC clones libraries, for instance, the CHORI-240 cattle library (e.g., on the basis of NCBI Bos_taurus_UMD_3.1.1 Primary Assembly data) or INRA library [64]. For FISH application, genomic BAC DNA is fluorescently labelled with biotin- 16-dUTP or digoxigenin-11-dUTP. In some specific cases, such as accurate rcps identification or evolutionary studies, combination of both BAC probes and WCP probes is used, sometimes complemented with array-comparative genomic hybridisation (array CGH). Array CGH is performed to confirm the potential association of reciprocal translocation with loss or gain of genetic material and to identify the presence copy number variations (CNVs) throughout the genome. In general, CNVs are linked with genomic rearrangements, including insertions or deletions (indels), duplications, inversions, and translocations [65]. They result from double-strand breaks that cannot be precisely repaired. In case of genome derived from individual cell, whole-genome amplification approach is required to ensure the availability of sufficient material for copy-number variation analysis [66]. Whole-genome amplification (WGA) is an advanced method allowing the preparation of large amounts of DNA required for extensive genotyping examinations.

The basis for these large-scale genotyping studies to identify genes that contribute to economically important traits became the bovine genome sequence project [67]. It was found that WGA is a suitable method for the amplification and recovery of DNA from bull semen samples for routine genomic investigation [67]. Except for degenerate oligonucleotide-primed polymerase chain reaction (DOP-PCR), other WGA approaches can be used, such as multiple displacement amplification (MDA) and multiple annealing and looping-based amplification cycles (MALBAC), overviewed in [65]. MDA provides much higher genome coverage than DOP-PCR but causes over amplification in certain genomic regions and under amplification in others. MALBAC is quasi-linear amplification (on the contrary to exponential amplification by DOP-PCR or MDA) that results in accuracy for CNV detection. The modern genomic approach is highly appreciated in human preimplantation genetic testing (PGT) of balanced translocations during assisted reproductive technology. Initially, fluorescence in-situ hybridisation (FISH) was used for PGT, with some technical limitations, such as ambiguous signals. Later, other methods, including array CGH (aCGH), SNP array, and whole-genome sequencing, were successfully applied for clear identification of embryos with chromosomally unbalanced translocation and aneuploidies. However, these techniques can hardly distinguish the balanced and structurally normal embryos. These problems were recently overcome by Zhang et al. [68], who developed a new method BasePhasing based on Infinium Asian Screening Assay-24v1,0 (ASA). Infinium ASA bead chip-based BasePhasing pipeline showed good performance in balanced translocation carrier testing in PGT.

Identification of rearrangements between cattle chromosomes (such as Robertsonian and reciprocal translocations) is also facilitated by the FISH method, which uses a set of subtelomeric probes on a multihybridisation device [52].

## 4. Cattle Chromosomal Aberrations in Genotoxicity Studies

In cattle-breeding practice, cytogenetic analysis is mostly used in connection with reproduction. The main aim is to prevent the adverse effect of chromosomal aberrations on reproductive efficiency. In addition to this employment of cytogenetics, the evaluation of cattle chromosomal aberrations can be a beneficial part of the complex battery of the tests for environmental genotoxicity assessment. This assessment can be performed in both in-vivo and in-vitro conditions. The purpose is to evaluate the level of structural changes on chromosomes (chromatid and chromosome breaks/exchanges) that might be induced by different kinds of environmental factors.

In the case of in vivo, the total exposure of the animals to the genotoxic factors of the polluted environment may be monitored by cytogenetic assay. However, it is difficult to determine the contribution of individual environmental factors to overall genotoxicity. As mentioned Rubeš et al. [69] animals reared in contaminated environments are directly exposed to these contaminants, and specifically herbivores may be exposed via polluted feed. Feeding cattle with roughage can increase the impact of the breeding site. As a result, cattle are directly affected by environmental toxins, or they are carriers of toxins in the human food chain [69]. For instance, some chemical agents have antinutritive properties, i.e., they reduce the nutritional value of the diet [1]. Concern arises for humans consuming cattle products (e.g., milk) after cattle have absorbed the chemicals presented in contaminated herbage in industrial zones. Milk and milk products are prone to be negatively influenced because of the lipophilic nature of some chemicals (e.g., dioxins) and the high content of fat in most milk products. Livestock can be accidentally poisoned by pesticides, such as fungicides applied to grains, potatoes, and other agricultural material [70], or by environmental contamination resulting from excessive use of chemicals in agriculture. The most frequently reported species are cattle; however, according to Guitart et al. [71], clinical cases of poisoning are only occasionally studied in depth. In environment, the presence of pesticide mixtures is common, with unpredictable effect because of complexity of toxicological interactions [72]. In these cases, a chromosomal aberration test can be the method of choice. An example should be dairy cattle exposed to genotoxic substances in a heavily polluted area. Cytogenetic analysis showed a significantly higher count of aberrant cells (peripheral lymphocytes) in comparison with animals in a normal environment [73]. Similarly, Rubeš et al. [69] found a highly significant difference in the frequency of aberrant cells of the industrial region of Pardubice in the Czech Republic (higher frequency) when compared with the agricultural area (lower frequency). The results indicated that industry in the Pardubice region (chemical industry, thermal power stations, oil processing) resulted in high exposure of farm animals to genotoxicants and a risk of food-chain contamination. It should also be noted that a variety of chemical compounds present in the environment at low doses is thought to affect reproductive functions in human and animals following prolonged exposure [74].

The recent experiment of Nakamura et al. [75] applied the lymphocytes from cattle grazing in the ex-evacuation zone of the nuclear power plant in Fukushima (FNPP) to in-vivo measuring of γ-H2AX foci. Detection of phosphorylated γ-H2AX foci is a sensitive quantitative assay based on visualization of these foci on the DNA break sites. This method is valuable for the evaluation of DNA double-strand breaks (DSBs) that are the most dangerous lesions, leading to loss of genetic information. The authors showed that DNA DSBs were significantly increased in cattle living in the FNPP evacuation zone. However, they recommend taking into consideration DNA damage-repair capacity and including more markers also containing chromosomal aberrations.

Chromosomal aberration test can also be applied under in-vitro conditions in genotoxicity studies. The standard in-vitro chromosomal aberration test allows the identification of specific agents that induce unstable structural chromosomal aberrations (chromatid and isochromatid breaks; chromatid and isochromatid exchanges) in cattle whole-blood cell cultures. Although their frequency decreases after the first cell cycle (24 h), they are a recognized indicator of the early effect of a given chemical substance. The procedure starts with sterile blood collection followed by culture of the cells in a medium (e.g., RPMI 1640 plus HEPES and L-glutamine) supplemented with bovine fetal serum, antibiotics, antimycotics, and a mitogen (phytohaemagglutinin or pokeweed). Mitogen is responsible for the stimulation of lymphocytes, which start to propagate by mitotic division. Treatment times with pesticides are performed for the last 24 h and 48 h of incubation. To stop division in the metaphase stage, colchicine or colcemid is usually added a few tens of minutes before the end of the culture. The cells are collected by centrifugation, hypotonized in 0.075 M KCl, and fixed in Carnoy solution (methanol/acetic acid). The microscopic slides are prepared by an air-dried method and stained with Giemsa dye. It should be noted that when compared with the situation in vivo, the natural metabolic transformation of some chemicals is absent in cultured cells in vitro. This is important because the intermediates of examined chemicals can be potentially reactive metabolites able to form DNA adducts. Therefore, the tests conducted in vitro should be performed not only without but also with an exogenous source of metabolic activation. This is usually achieved through the addition of rodent liver-metabolizing systems (S9 fraction) to the cell cultures. S9 fraction usually contains both phases I enzymes able to activate mutagens and phase II detoxifying enzymes.

**Figure 4:**
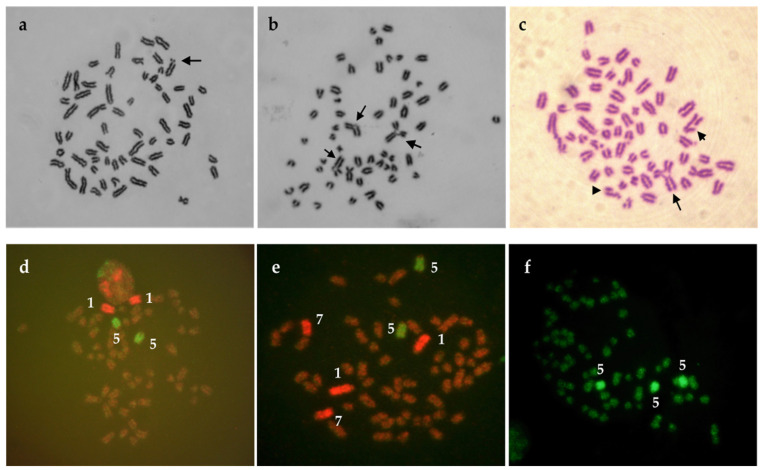
Bull metaphases exposed to pesticides in vitro. (**a**) Isochromatid break after exposure to tebuconazole/prothioconazole-based fungicide formulation indicated by an arrow (**b**) Chromatid exchanges after exposure to tebuconazole-based fungicide marked with three separate arrows (**c**) Chromatid exchange (the longest arrow), breaks (shorter arrow and arrowhead), and fragmentation (many breaks in the metaphase) after tebuconazole-based fungicide exposure. (**d**) Normal metaphase hybridised with WCP probes BTA 1 and BTA 5. (**e**) Normal metaphase hybridised with WCP probes BTA 1, BTA 5, and BTA 7. (**f**) Aneuploidy. Three copies of chromosome 5 (BTA 5) are visible in the metaphase plate. Metaphase originates from cultures treated with tebuconazole-based fungicide formulation.

Cattle cell cultures seems to be suitable for testing the genotoxic effects of chemical agents (e.g., pesticides) as has been shown in works of Lioi et al. [76], Rossi et al. [77], Schwarzbacherova et al. [78], Šivikova et al. [79], and recently Ferré et al. [80]. The authors studied genotoxicity induced by different types of pesticides in bovine lymphocyte cultures in vitro and suggested possible genotoxic effect of glyphosate, vinclozolin, DPX-E9636 [76], fungicide formulation containing epoxiconazole and fenpropimorph [78], pure epoxiconazole [79], as well as cypermethrin, chlorpyrifos, and their subsequent mixture [80]. In addition, an increased level of breaks was observed after exposure of cattle cells to triazole pesticide formulation (tebuconazole/prothioconazole) [81] (Figure 4a) and tebuconazole-based fungicide formulation (Figure 4b,c) [82].

To complete conventional Giemsa cytogenetic analysis, fluorescence in-situ hybridisation (FISH) with whole-chromosome painting probes (FISH-WCP) (Figure 4d–f) can be used as the method of choice. WCP probes for specific BTA chromosomes enable visualization of stable chromosomal aberrations (one-way translocations, reciprocal translocations, and insertions) that are not visible after Giemsa staining of chromosomes, but various agents induce them [54]. For the assessment of the mutagenic impact of chemical agents by FISH, the knowledge about the spontaneous (basal) frequency of the different translocations types is important. This frequency was determined in humans, cows, and pigs by Rezacova et al. [59] using dual-coloured FISH with WCP probes. Surprisingly, cows showed a much lower frequency of total translocations in comparison with humans and pigs. The authors supposed that results might be related to the lowest proportion of the painted genome in cattle when compared with the proportion of the painted genome in humans and pigs. They also assumed that cattle might have a reduced sensitivity to the chromosomal mechanisms, which can cause structural chromosomal aberrations. This statement could be probably supported by the recent knowledge indicating that cows (also sheep, rabbits, and chickens) have a high proportion of peripheral gamma-delta T cells. A high number of gamma-delta T cells is required to rapid handle the high burden of bacterial, viral, and fungal pathogens these animals are exposed to in their environment [83].

In case of evaluation of bendiocarbamate effect on bovine lymphocytes [61], FISH with two different whole-chromosome painting probes (BTA1 and BTA5) was used for the complementation of conventional chromosomal analysis. Only very low frequency of one-way translocations was detected in cells of two healthy bull donors. More painting probes for bovine chromosomes BTA 1, 5, and 7 were used to assess the effect of triazole pesticide formulation; nevertheless, no stable aberrations were observed [81]. The same probes were applied by Drážovská et al. [84] for epoxiconazole- and fenpropimorph-based fungicide in bovine lymphocytes in vitro. Similarly, no translocations were detected in treated cells. The potential genotoxic effect of thiacloprid formulation on bovine peripheral lymphocytes was evaluated by Galdíková et al. [85] using the comet assay and the cytogenetic endpoints: chromosome aberrations (CAs), sister chromatid exchanges (SCEs), micronuclei (MNi), and FISH using three whole-chromosome painting probes for bovine chromosomes 1, 5, and 7 (BTA1, BTA5, and BTA7). Additionally, in this case, the presence of stable aberrations (translocations) was not recorded. The results of all the above-mentioned experiments can be explained by many reasons. One of them is the relatively low proportion of painted genome (10.35% in case of BTA1 and BTA5; 14.53% in case of BTA1, BTA5, and BTA7). This situation can be partially compensated by the high number of bovine cells analysed per individual. However, obtaining a sufficient number of cells in routine practice is difficult because of insufficient stimulation of lymphocyte proliferation by mitogen phytohemagglutinin (PHA). Therefore, a combination of PHA with pokeweed mitogen (or pokeweed mitogen alone) should be used in cattle obtaining a higher mitotic index. Moreover, in FISH-WCPs, experiments are also important to carefully considering the chemical structure and mechanism of pesticide action, dose, and time of treatment. This approach was explained by Marshall and Obe [86], who stated that different pathways are followed during the production of breaks and exchanges by various chemicals. The authors suggested that the value of applying chromosome painting (WCP probes) to clastogenicity testing can be improved by the possibility to paint the greater part (or all) of the genome to be examined for translocations. Despite some of the disadvantages mentioned in cattle, fluorescence staining of bovine chromosomes with whole-chromosome painting probes is an important adjunct to the conventional cytogenetic method in genotoxicity studies.

In genotoxicology and toxicogenomics, array CGHs have also been employed. Chromosomal and array CGH is being demonstrated to be an effective tool for investigating copy-number changes (variations) in the whole genome, DNA expression patterns, as well as loss of heterozygosity after the genotoxic impact [87]. Nowadays, advanced cytogenetic analysis can be conducted using whole-genome array platforms or next-generation sequencing (NGS).

## 5. Conclusions

For the detection of chromosomal aberrations in cattle, cytogenetic analysis, especially using advanced FISH techniques, is a valuable tool. In the review, we focused mostly on the translocations (Robertsonian and reciprocal) that are in cattle connected with reproductive-related problems, such as impaired fertility and embryonal mortality. In general, fertility reduction in carrier heterozygous bulls is caused by the production of unbalanced gametes during meiosis and consequently by the creation of a part of embryos with a high probability of dying. For this reason, a routine cytogenetic evaluation of bulls, particularly the ones intended to use in artificial insemination, should be recommended to be conducted as one of the solutions. The close collaboration of breeders with veterinarians might be helpful in the prevention of economic consequences caused by using improper animals in reproduction. In addition, we reported in this review that bovine lymphocytes can be successfully used in an in-vitro chromosomal aberration assay in genotoxicity studies.

## Data Availability

Data sharing is not applicable to this article as no new data were created in this study.
